# Peripheral immune phenotyping identifies an immunosuppressed sepsis endotype with potential relevance for immune stratification

**DOI:** 10.3389/fimmu.2026.1939205

**Published:** 2026-09-04

**Authors:** Rong Luo, Yixia Xie, Xiaofan Deng, Chengying Hong, Yingying Liang, Jinquan Xia, Da Zhang, Jingjin Liu, Chunbo Chen, Huaisheng Chen

**Affiliations:** 1 The Second Clinical Medical College of Jinan University, Department of Critical Care Medicine, Shenzhen People’s Hospital, Shenzhen, Guangdong, China; 2 Department of Clinical Medical Research Center, Shenzhen People’s Hospital (The First Affiliated Hospital, Southern University of Science and Technology; The Second Clinical Medical College, Jinan University), Shenzhen, Guangdong, China; 3 Department of Critical Care Medicine, Shenzhen People’s Hospital (The Second Clinical Medical College, Jinan University; The First Affiliated Hospital, Southern University of Science and Technology), Shenzhen, Guangdong, China; 4 Department of Geriatrics, Shenzhen People’s Hospital (The First Affiliated Hospital, Southern University of Science and Technology; The Second Clinical Medical College, Jinan University), Shenzhen, Guangdong, China; 5 Guangdong Provincial Clinical Research Center for Geriatrics, Shenzhen Clinical Research Center for Geriatrics, Department of Geriatrics, Shenzhen People’s Hospital, the First Affiliated Hospital, Southern University of Science and Technology; The Second Clinical Medical College of Jinan University, Shenzhen, China

**Keywords:** HLA-DR, immune phenotyping, immune suppression, lactate, precision immunotherapy, sepsis

## Abstract

**Background:**

Sepsis is characterized by profound immune dysregulation, yet the biological significance and clinical application of peripheral immune phenotyping in sepsis remain incompletely defined. This study aimed to define internally identified immune phenotypes in sepsis and evaluate their prognostic and translational relevance.

**Methods:**

In this single-centre retrospective cohort study, 374 adult patients with sepsis or severe infection were included. Six peripheral immune markers (total T cells, CD4^+^ T cells, CD8^+^ T cells, total B cells, NK cells, and monocyte HLA-DR) together with clinically relevant laboratory indicators were analysed to characterize immune and clinical-laboratory heterogeneity in sepsis. Internal validity metrics were used to determine the optimal cluster number. The prognostic value of immune phenotypes was benchmarked against routine laboratory indicators through exhaustive combinatorial clustering and repeated cross-validated logistic regression. Associations with illness severity (SOFA, APACHE II, and NUTRIC) were further assessed.

**Results:**

Two internally stable immune phenotypes were identified: an immune-preserved phenotype (n = 306) and an immune-suppressed phenotype (n = 61). The immune-suppressed phenotype exhibited globally reduced lymphocyte subsets and lower monocyte HLA-DR, accompanied by lower platelet and white blood cell counts, higher inflammatory markers, and slightly higher SOFA scores. However, in-hospital mortality did not differ significantly between phenotypes (39.0% vs 30.6%, *P* = 0.224). Immune phenotyping achieved limited prognostic separation (Δ = 8.9 percentage points), substantially lower than laboratory-based combinations (Δ = 63.3 points). In predictive modelling, the six-marker immune model showed the weakest discrimination (AUC = 0.646 ± 0.066), whereas SOFA alone outperformed all six-marker combinations (AUC = 0.747 ± 0.055). Lactate, as a severity-associated laboratory indicator, demonstrated a stronger prognostic association than immune parameter suggesting that immune suppression and physiological stress represent complementary dimensions of sepsis pathobiology.

**Conclusion:**

Peripheral immune phenotyping identifies biologically distinct immune-preserved and immune-suppressed states in sepsis. Although these immune phenotypes provide limited additional prognostic discrimination beyond established severity scores and routine laboratory indicators, they provide valuable insights into immune dysfunction and represent a potential framework for immune stratification. Further longitudinal studies are needed to determine whether these immune phenotypes can inform future personalized immunomodulatory strategies.

## Introduction

Sepsis is a major global health burden and a leading cause of death in critically ill patients, affecting an estimated 49 million people annually and contributing to approximately 11 million deaths worldwide, accounting for nearly 20% of all global deaths (1, 2). It is defined as life-threatening organ dysfunction caused by a dysregulated host response to infection, as established by the Sepsis-3 consensus (3, 4). Despite antimicrobial therapy development, organ-support strategies, and critical care management, sepsis continues to impose a substantial clinical and economic burden worldwide (US$24 billion per year in the United States alone) (5). Hospital mortality remains high, ranging from 20% to 40%, and can exceed 50% in patients with septic shock or multiple organ failure (6). A major challenge in sepsis management is the heterogeneity of host immune dysfunction, which contributes to variable clinical trajectories and undermines the effectiveness of standardized therapeutic approaches. The pathobiology of sepsis involves a highly dynamic and dysregulated immune response, in which hyperinflammation and immunosuppression coexist and collectively shape disease progression and clinical outcomes (7–10). Recent translational studies have further highlighted the contribution of regulated cell-death pathways, including NETosis and pyroptosis, to immune dysregulation and organ injury during sepsis (11). Although the early phase of sepsis is often characterized by an excessive release of pro-inflammatory cytokines-tumor necrosis factor-α (TNF-α), interleukin (IL)-1β, and IL-6, many patients subsequently develop immunoparalysis (9), a state marked by lymphocyte depletion, impaired antigen presentation, reduced monocyte HLA-DR expression, and functional exhaustion of innate and adaptive immune cells (12, 13). This immunosuppressive phase is associated with increased susceptibility to secondary infections, prolonged ICU stay, and poor clinical outcomes (14). Beyond immune-cell dysfunction, sepsis involves complex alterations in host physiology, including disturbances in inflammatory responses and systemic metabolic homeostasis. Elevated lactate reflects systemic physiological stress in sepsis and may result from multiple mechanisms, including impaired tissue perfusion, inflammation, and altered metabolic processes. During sepsis progression, immune cells undergo profound metabolic alterations characterized by mitochondrial dysfunction, impaired oxidative phosphorylation, enhanced glycolytic dependence, and altered substrate utilization (15). Lactate accumulation reflects not only tissue hypoperfusion but also inflammatory metabolic remodeling and mitochondrial stress. These metabolic changes influence immune-cell differentiation, cytokine production, and functional exhaustion. Therefore, integrating immune phenotyping with routinely available clinical-laboratory indicators may provide a more comprehensive assessment of sepsis heterogeneity and help generate hypotheses for future immune-stratification studies.

Recent efforts to characterize sepsis heterogeneity have led to the identification of distinct biological endotypes using transcriptomic, proteomic, and immunological profiling approaches. Several transcriptomic classifications-Sepsis Response Signature (SRS) and Molecular Diagnosis and Risk Stratification (MARS) endotypes, have demonstrated that immune suppression represents a major subtype of sepsis (16). Similarly, Flow-cytometry-based studies have identified reduced monocyte HLA-DR expression and alterations in lymphocyte subsets as key hallmarks of sepsis-induced immune dysfunction, with emerging evidence supporting their role as biomarkers for immune stratification and therapeutic selection (17, 18). Despite growing evidence supporting immune dysregulation as a central determinant of sepsis progression, the clinical utility of peripheral immune phenotyping remains inadequately defined. Although biomarkers such as monocyte HLA-DR expression and lymphocyte subset count provide important insights into host immune competence, their incremental prognostic value beyond established severity scores and conventional laboratory indicators remains uncertain. Defining this distinction is clinically important, as the principal value of immune phenotyping may lie less in mortality prediction and more in identifying immunoparalysis-associated endotypes suitable for targeted immunomodulatory interventions. In the present study, we applied unsupervised clustering to six peripheral immune markers in a retrospective cohort of patients with sepsis or severe infection to identify internally stable immune phenotypes. We further evaluated whether these immune-defined phenotypes were associated with clinically relevant outcomes and benchmarked their prognostic performance against routine laboratory variables and established illness-severity scores. Importantly, mortality analysis was performed not to assume immune phenotypes as mortality predictors, but to determine whether immune endotypes provide additional clinical information beyond conventional severity assessment. We hypothesized that immune phenotyping would identify biologically coherent host-response endotypes, while its primary value would lie in biological stratification rather than standalone mortality prediction.

## Materials and methods

### Study design and ethical approval

This single-center retrospective cohort study was conducted in accordance with the principles of the Declaration of Helsinki and relevant institutional regulations. The study protocol was reviewed and approved by the Clinical Research Ethics Committee of Shenzhen People’s Hospital, Shenzhen, China (Approval No. LL-KY-2026047–01 and LL-KY-2024136-01). written informed consent was obtained from all participants or their legally authorized representatives.

### Study population

**Figure 1:**
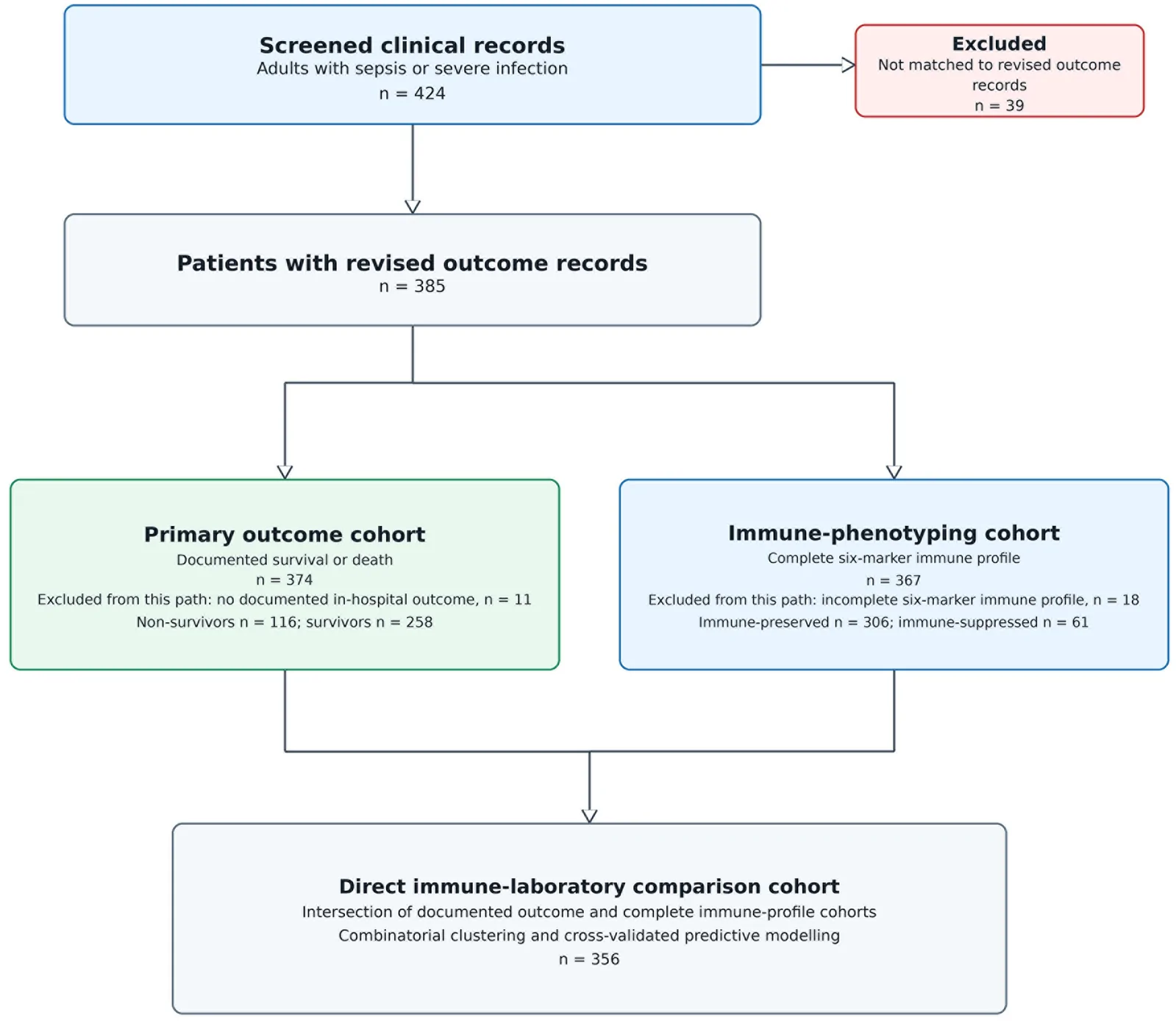
Flow diagram of patient selection and analytical cohorts.

Adult patients (≥18 years) admitted to Shenzhen People’s Hospital with sepsis or severe infection between February 2025 and May 2026 were retrospectively screened. Sepsis was defined according to the Sepsis-3 criteria as life-threatening organ dysfunction caused by a dysregulated host response to infection (3). Patients classified as having severe infection without fulfilling Sepsis-3 criteria were defined as individuals with clinically suspected or microbiologically confirmed infection requiring hospital admission and intensive monitoring due to systemic inflammatory response, organ dysfunction risk, or need for advanced supportive care, but without documented sepsis-related organ dysfunction at assessment. These patients were included because immune profiling was performed clinically in patients with suspected severe infection or sepsis and allowed evaluation of immune alterations across the spectrum of severe infectious illness. Eligible patients were required to have a documented in-hospital outcome (survival or death) and at least one baseline immune assessment performed within 24 hours of admission. Sepsis classification was determined using clinical information available at the time of admission and immune sampling. The screening and selection process, including excluded patients and reasons for exclusion, is summarized in **Figure 1**. Immune profiling was performed as part of routine clinical immune-status assessment in patients with suspected severe infection or sepsis according to institutional practice, rather than being determined by study investigators. Patients with incomplete outcome data, missing key immune variables, hematological malignancies, ongoing immunosuppressive therapy, end-stage autoimmune disease, or active chemotherapy were excluded. Among 385 patients with revised clinical records, 374 had documented in-hospital outcomes and were included in the primary outcome cohort. Separately, 367 patients had complete six-marker immune profiles and were included in immune-phenotyping analyses. Direct comparative analyses between immune and laboratory variables were restricted to 356 patients with complete datasets for both immune and laboratory variables.

### Clinical data collection

Demographic, clinical, laboratory, treatment, and outcome data were extracted from electronic medical records using a standardized data abstraction protocol. Baseline variables included age, sex, illness-severity scores (APACHE II, SOFA, and NUTRIC), vital signs (temperature, heart rate, and mean arterial pressure), and oxygenation status (PaO_2_/FiO_2_ ratio). Laboratory variables included glucose, albumin, blood urea nitrogen (BUN), creatinine, platelet count, lactate, hemoglobin, total bilirubin, red blood cell distribution width-coefficient of variation (RDW-CV), procalcitonin (PCT), interleukin-6 (IL-6), C-reactive protein (CRP), white blood cell (WBC) count, neutrophil percentage, lymphocyte percentage, and monocyte percentage. Treatment-related variables included duration of mechanical ventilation, thymosin use, and ulinastatin use. The primary clinical outcome was in-hospital mortality. Information regarding infection source, microbiological findings, septic shock status, major comorbidities, and relevant treatments before immune sampling was collected when available and considered in the interpretation of immune phenotypes.

### Immune marker assessment

Peripheral blood samples for immune profiling were collected within 24 hours of hospital admission whenever possible. Because this was a retrospective real-world cohort in which immune assessment was performed as part of routine clinical practice, the exact temporal relationship between immune sampling and all therapeutic interventions could not be completely standardized. Immune profiling was performed prior to initiation of immunomodulatory therapy whenever feasible. Six predefined immune markers were analyzed: total T-cell count, CD4^+^ T-cell count, CD8^+^ T-cell count, total B-cell count, natural killer (NK) cell count, and monocyte human leukocyte antigen-DR (HLA-DR) expression. Peripheral blood samples were collected in EDTA-anticoagulated tubes and processed according to standardized institutional laboratory protocols. Peripheral blood immune profiling was performed using multiparameter flow cytometry according to institutional standard operating procedures. Samples were analyzed using a DxFLEX flow cytometer (Beckman Coulter, USA). Immune-cell subsets were identified using a predefined antibody panel targeting CD3^+^ T cells, CD3^+^CD4^+^ T cells, CD3^+^CD8^+^ T cells, CD19^+^ B cells, CD3^-^CD16^+^CD56^+^ NK cells, and CD14^+^HLA-DR^+^ monocytes. Detailed antibody information, including antibody clones and fluorochromes, is provided in **Supplementary Table 1**. Flow cytometry compensation was performed using single-stained controls according to manufacturer recommendations. Instrument performance and analytical quality control were routinely assessed before sample acquisition. Data acquisition and analysis were performed using standardized gating strategies established by the clinical laboratory. Representative gating strategies for immune-cell identification and monocyte HLA-DR analysis are provided in **Supplementary Figures 1**, **S2**. Monocyte HLA-DR expression was quantified as the percentage of HLA-DR-positive monocytes after gating on the monocyte population. Monocytes were identified based on standard forward/side scatter characteristics and CD14 expression, followed by assessment of HLA-DR positivity within the gated monocyte population. HLA-DR positivity was defined according to the laboratory-established fluorescence threshold using appropriate controls. The same analytical criteria were consistently applied across all samples.

### Data preprocessing

To ensure comparability across analyses, variables with right-skewed distributions, including immune cell counts and selected laboratory indicators (lactate, total bilirubin, PCT, IL-6, CRP, creatinine, and BUN), were log-transformed using log(1+x). Variables with missingness exceeding 20% were excluded from downstream analyses. For variables with lower missingness, missing values were imputed using median imputation to preserve sample size and minimize bias in unsupervised analyses. All continuous variables were standardized to zero mean and unit variance before clustering and predictive modelling. To avoid information leakage, all preprocessing steps within predictive models were fitted independently within each cross-validation fold.

### Identification of immune phenotypes

Unsupervised K-means clustering was applied to the six immune markers to identify distinct immune phenotypes. K-means was selected for its robustness, interpretability, and suitability for standardized continuous immunological variables. Because several immune markers represent related lymphocyte populations, potential redundancy among clustering variables was considered during interpretation. Variables were standardized before clustering to ensure equal numerical scaling, and cluster interpretation was based on the coordinated pattern across all six immune markers rather than any single variable. In addition, principal component analysis was performed to evaluate the contribution structure of individual immune variables. Clustering was performed with 100 random initializations (n_init = 100) and fixed random seeds to improve stability and reduce local-minimum bias. The optimal number of clusters (k = 2–6) was determined using six internal validation metrics: silhouette coefficient, Calinski–Harabasz index, Davies–Bouldin index, bootstrap-adjusted Rand index, Gaussian mixture model Bayesian information criterion (GMM-BIC), and gap statistic. The optimal cluster number was determined by integrating internal validation metrics with biological interpretability and cluster stability considerations. Although different validation metrics showed partially discordant preferences, the k=2 solution was supported by the silhouette coefficient, Calinski–Harabasz index, and Davies–Bouldin index, which collectively indicate improved separation and compactness of clusters. Higher-order solutions generated additional subdivisions but resulted in less biologically interpretable immune patterns and smaller subgroups. Therefore, a two-cluster solution was selected as the most clinically interpretable representation of major immune states, and clusters were classified as immune-preserved and immune-suppressed according to their immune-marker profiles.

### Exhaustive combinatorial clustering analysis

To benchmark the prognostic resolution of immune phenotyping against routine laboratory variables, exhaustive combinatorial clustering was performed across all possible variable combinations of size 2–6. Analyses were conducted separately for the laboratory-only variable pool (16 indicators) and the combined laboratory-plus-immune pool (22 indicators). For each combination, patients were partitioned into three clusters using K-means clustering (k = 3). This framework was selected to explore prognostic heterogeneity while maintaining sufficient subgroup size for mortality estimation. Because cluster number influences mortality separation, comparisons of mortality spread across clustering frameworks with different numbers of clusters were interpreted as exploratory rather than direct quantitative comparisons. Only clusters containing at least 15 patients per subtype were considered valid. Prognostic resolution was quantified as the difference in in-hospital mortality between the highest-risk and lowest-risk clusters (death-rate spread, Δ). Because exhaustive optimization may introduce optimistic selection bias, the maximum observed Δ was interpreted as an upper-bound estimate rather than an unbiased effect size.

### Frequency and enrichment analysis

To identify variables contributing most strongly to prognostic separation, significant variable combinations (*P* < 0.05) underwent frequency and enrichment analysis. Frequency was defined as the number of times a variable appeared among the top-performing combinations. Enrichment was defined as the difference in mean death-rate spread (Δ) between combinations containing versus excluding that variable, thereby quantifying its incremental prognostic contribution.

### Predictive modelling

Logistic regression models were constructed to predict in-hospital mortality using six predefined predictor sets: the six immune markers alone; the best-performing laboratory marker combination; the same laboratory combination with natural killer (NK) cells substituted; the SOFA score alone; all 22 combined immune and laboratory indicators; and the SOFA score combined with all 22 indicators. The laboratory marker combination was selected based on its prognostic separation performance in the exploratory combinatorial clustering analysis before predictive modelling. Therefore, this feature selection step was considered exploratory, and the resulting predictive estimates were interpreted accordingly. Model performance was evaluated using repeated stratified 5-fold cross-validation with 20 repetitions to improve robustness and reduce variance. Discriminatory ability was assessed using the area under the receiver operating characteristic curve (AUC). Pairwise comparisons of AUCs were performed using the DeLong test, implemented according to the fast algorithm described by Sun and Xu (19).

### Association between immune phenotype and illness severity

Associations between immune phenotypes and illness-severity scores (SOFA, APACHE II, and NUTRIC) were evaluated using both dichotomized and continuous approaches. Median-split comparisons were performed using Fisher’s exact test. Univariable logistic regression was used to estimate odds ratios (ORs) and 95% confidence intervals (CIs) for the immune-suppressed phenotype.

### Statistical analysis

Continuous variables are presented as mean ± standard deviation or median (interquartile range), depending on distribution. Group comparisons were performed using Welch’s t-test or Mann–Whitney U test as appropriate. Categorical variables were compared using Fisher’s exact test. All statistical tests were two-sided, and *P* < 0.05 was considered statistically significant. No adjustment for multiplicity was applied for descriptive analyses. Statistical analyses were performed using Python (pandas, NumPy, SciPy, scikit-learn, and statsmodels), with fixed random seeds to ensure reproducibility. Normality was assessed using the Shapiro–Wilk test together with visual inspection of distribution plots.

## Results

### Cohort characteristics and association with in-hospital mortality

A total of 374 patients with sepsis or severe infection and documented in-hospital outcomes were included in the study, of whom 116 (31.0%) died during hospitalization and 258 (69.0%) survived. All 374 patients included in the primary outcome cohort fulfilled the Sepsis-3 criteria (100%). Among the overall cohort, 206 (55.1%) had septic shock. Baseline demographic, clinical, laboratory, immune, and treatment characteristics stratified by survival status are summarized in **Table 1**. No significant differences were observed between survivors and non-survivors in age (65.12 ± 15.21 vs 66.79 ± 15.59 years, *P* = 0.334) or sex distribution (*P* = 0.470). In contrast, illness-severity scores demonstrated marked separation between outcome groups. Non-survivors had significantly higher APACHE II (28.58 ± 7.46 vs 23.02 ± 7.38, *P* < 0.001), SOFA (11.02 ± 3.39 vs 7.97 ± 3.04, *P* < 0.001), and NUTRIC scores (7.47 ± 1.87 vs 6.02 ± 1.94, *P* < 0.001). Among physiological and laboratory parameters, non-survivors exhibited significantly lower mean arterial pressure, reduced PaO_2_/FiO_2_ ratio, higher lactate levels, lower platelet counts, lower hemoglobin, higher BUN, higher RDW-CV, elevated total bilirubin, and mildly increased creatinine (all *P* < 0.05). Notably, inflammatory markers including PCT, IL-6, and CRP did not significantly differ between groups. Among the six immune markers, only monocyte HLA-DR (42.02 ± 24.19% vs 56.08 ± 25.24%, *P* < 0.001), CD8^+^ T-cell count (*P* = 0.019), and NK-cell count (*P* = 0.016) were significantly lower in non-survivors, whereas total T cells, CD4^+^ T cells, and total B cells showed no significant differences. Non-survivors also required longer mechanical ventilation and received thymosin more frequently, likely reflecting confounding by indication.

**Table 1:** Baseline demographic, clinical, laboratory, immune, and treatment characteristics of the study cohort stratified by in-hospital outcome.

Variable	Overall (n=374)	Non-survivors (n=116)	Survivors (n=258)	Missing	Test	Statistic	*P* value
Demographics							
Age (years)	65.64 ± 15.33	66.79 ± 15.59	65.12 ± 15.21	0.5%	Welch t	-0.97	0.334
Sex (F/M)	117/257	33/83	84/174	0.0%	Fisher		0.470
Severity scores							
APACHE II	24.74 ± 7.83	28.58 ± 7.46	23.02 ± 7.38	0.0%	Welch t	6.69	<0.001
SOFA	8.91 ± 3.45	11.02 ± 3.39	7.97 ± 3.04	0.0%	Welch t	8.31	<0.001
NUTRIC	6.47 ± 2.03	7.47 ± 1.87	6.02 ± 1.94	0.0%	Welch t	6.86	<0.001
Vital signs							
Temperature(°C)	36.8	36.7	36.8	0.3%	Mann U	-0.97	0.331
Heart rate(beats/min)	100.96	107.52	98.02	0.0%	Welch t	3.18	0.002
MAP(mmHg)	64.0	58.5	65.0	0.0%	Mann U	-4.43	<0.001
Laboratory indicators							
PaO2/FiO2	265.19	243.45	274.96	0.0%	Welch t	-2.37	0.018
Glucose(mmol/L)	9.42	9.72	9.29	0.0%	Welch t	1.13	0.259
Albumin(g/L)	30.0	31.0	30.0	0.0%	Mann U	1.59	0.111
BUN(mmol/L)	11.1	12.9	9.9	0.0%	Mann U	3.30	<0.001
Creatinine(umol/L)	117.0	133.5	109.0	0.0%	Mann U	1.99	0.046
Bilirubin(umol/L)	20.0	25.5	18.3	0.0%	Mann U	2.59	0.010
Platelets(×10^9^/L)	121.0	91.0	134.5	0.0%	Mann U	4.37	<0.001
Lactate(mmol/L)	1.6	2.2	1.5	0.3%	Mann U	5.61	<0.001
Hemoglobin(g/L)	92.97	86.67	95.81	0.0%	Welch t	-3.64	<0.001
Immune indicators							
CD8+ T cells(cells/uL)	126.0	103.5	141.8	2.9%	Mann U	-2.35	0.019
NK cells(cells/uL)	78.9	63.6	88.6	2.9%	Mann U	-2.42	0.016
Monocyte HLA-DR(%)	51.62	42.02	56.08	4.0%	Welch t	-5.06	<0.001
Treatment							
Mechanical ventilation	2.0	8.0	1.0	3.2%	Mann U	6.33	<0.001
Thymosin use	259/103	67/47	192/56	3.2%	Fisher		<0.001
Ulinastatin use	269/93	80/34	189/59	3.2%	Fisher		0.245

Data are presented as mean ± standard deviation or median (interquartile range), unless otherwise indicated. Categorical variables are presented as number (percentage). APACHE II, Acute Physiology and Chronic Health Evaluation II; SOFA, Sequential Organ Failure Assessment; NUTRIC, Nutrition Risk in the Critically Ill; MAP, mean arterial pressure; BUN, blood urea nitrogen; PaO_2_/FiO_2_, arterial oxygen partial pressure/fraction of inspired oxygen ratio; RDW-CV, red blood cell distribution width coefficient of variation; PCT, procalcitonin; IL-6, interleukin-6; CRP, C-reactive protein; NK, natural killer; HLA-DR, human leukocyte antigen-DR. Continuous variables were compared using Welch’s t-test or Mann–Whitney U test, and categorical variables using Fisher’s exact test, as appropriate.

### Identification of internally stable immune phenotypes

**Figure 2:**
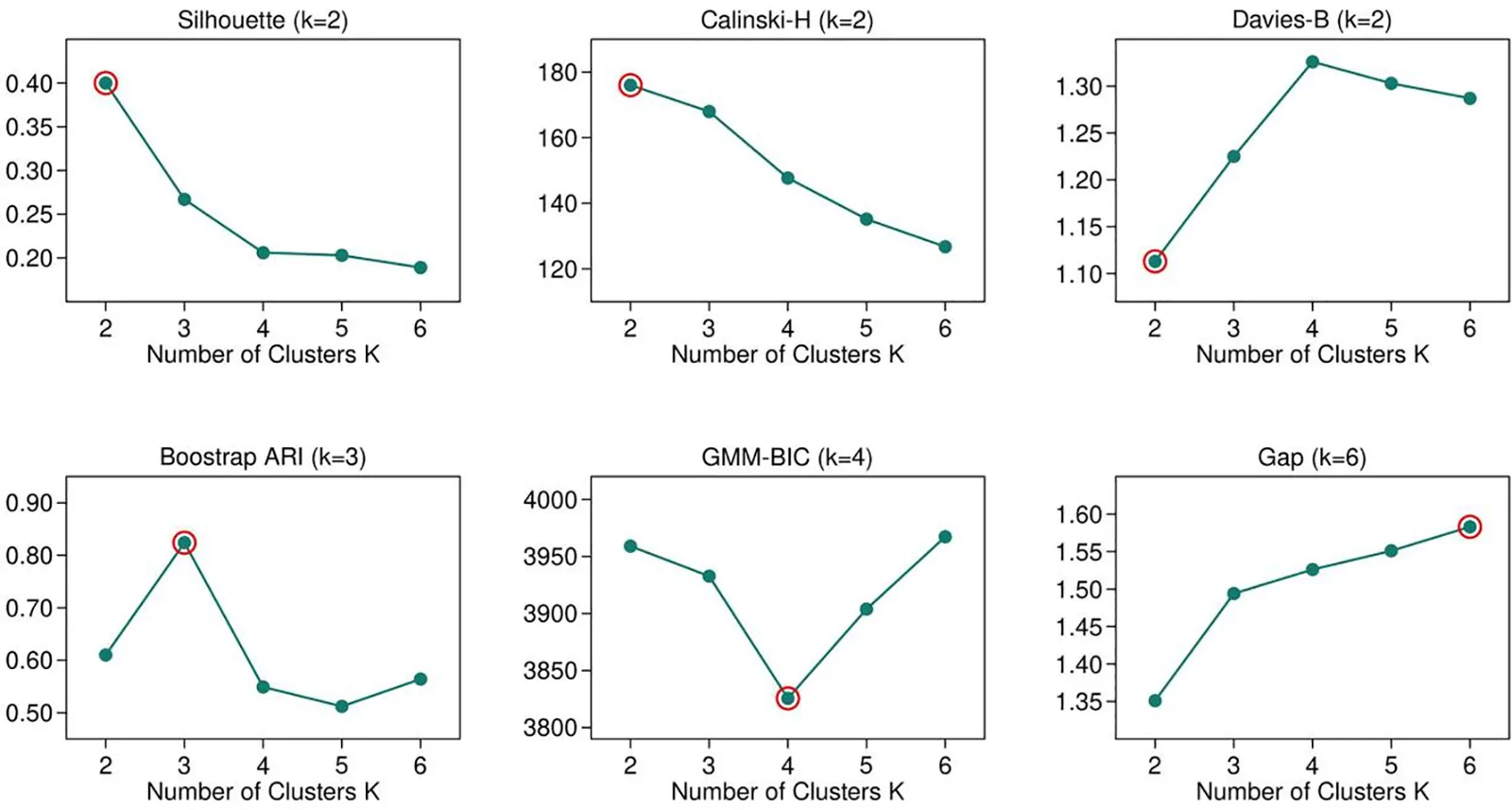
Identification of stable immune phenotypes using multidimensional immune-marker clustering. Internal validation metrics (silhouette coefficient, Calinski–Harabasz index, Davies–Bouldin index, bootstrap adjusted Rand index, GMM-BIC, and gap statistic) supported a two-cluster solution, defining immune-preserved and immune-suppressed sepsis phenotypes.

**Figure 3:**
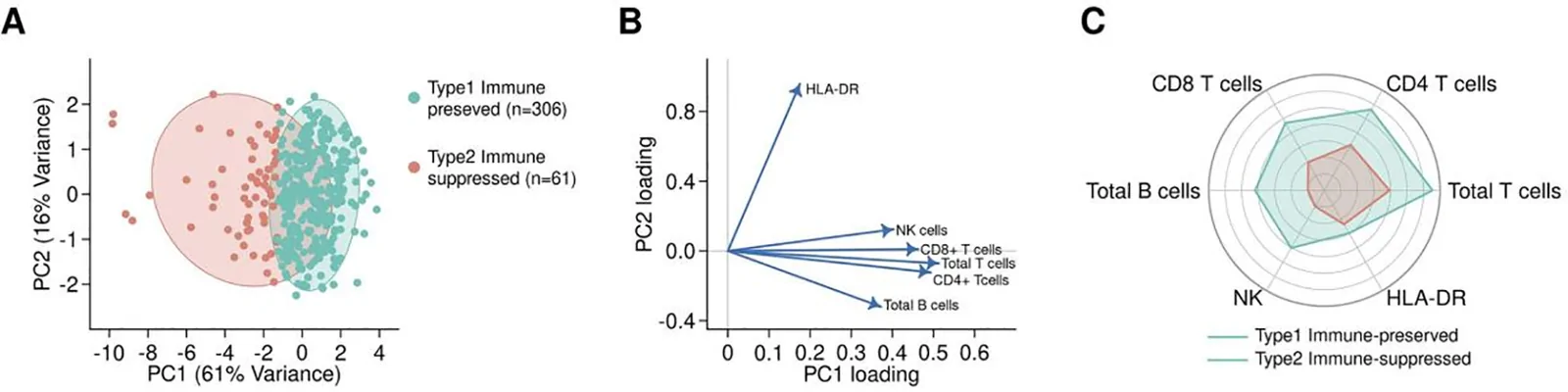
Immune-cell landscape defining immune-preserved and immune-suppressed sepsis phenotypes. **(A)** Principal component analysis (PCA) of the six peripheral immune markers shows separation between the immune-preserved phenotype (Type 1, n = 306) and the immune-suppressed phenotype (Type 2, n = 61). PC1 and PC2 explained 61% and 16% of the total variance, respectively. **(B)** PCA loading plot showing the contribution of individual immune markers to PC1 and PC2. Lymphocyte-related variables contributed predominantly to PC1, whereas monocyte HLA-DR showed a distinct loading pattern along PC2. **(C)** Radar plot of immune-marker profiles across the two phenotypes. The immune-suppressed phenotype showed coordinated reductions in total T cells, CD4^+^ T cells, CD8^+^ T cells, total B cells, NK cells, and monocyte HLA-DR expression compared with the immune-preserved phenotype.

Unsupervised K-means clustering of six peripheral immune markers identified two internally stable immune phenotypes. Cluster-number optimization using six internal validation metrics showed partial disagreement among metrics. The silhouette coefficient, Calinski–Harabasz index, and Davies–Bouldin index supported a two-cluster solution, whereas bootstrap ARI, GMM-BIC, and gap statistic favored higher cluster numbers. Considering the balance between statistical separation, cluster interpretability, and biological relevance, the two-cluster solution was selected as the most appropriate representation of major immune-response states (**Figure 2**). Principal component analysis demonstrated clear separation of the two clusters, with principal components 1 and 2 explaining 61% and 16% of total variance, respectively (**Figure 3A**). Based on their median immune marker profiles, the two clusters were designated as immune-preserved (n = 306) and immune-suppressed (n = 61, 16.6%). PCA demonstrated that lymphocyte-related variables contributed substantially to PC1 (61% of variance), reflecting the strong biological correlation among circulating lymphocyte subsets. However, monocyte HLA-DR showed a distinct loading pattern on PC2, indicating an additional dimension of immune regulation beyond lymphocyte abundance alone. The immune-suppressed phenotype was characterized not only by reduced lymphocyte populations but also by reduced monocyte HLA-DR expression, supporting a broader immune-dysfunction pattern rather than isolated lymphopenia (**Figure 3B**). Radar plots confirmed a consistent reduction across all six immune markers in the immune-suppressed phenotype (**Figure 3C**).

### Clinical characteristics of immune phenotypes

Comparisons between immune phenotypes are summarized in **Table 2**. By definition, the immune-suppressed phenotype exhibited significantly lower levels of all six immune markers (all *P* < 0.001). Beyond immune variables, this phenotype also demonstrated distinct systemic alterations, including lower platelet counts, lower WBC counts, lower monocyte and lymphocyte percentages, and higher neutrophil percentages (all *P* < 0.01). Markers of systemic inflammation, including PCT, IL-6, and CRP, were significantly higher in the immune-suppressed group, supporting a biologically coherent immunoparalysis-associated inflammatory state. SOFA scores were modestly higher in the immune-suppressed phenotype (10.07 vs 8.73, *P* = 0.013), whereas APACHE II and NUTRIC scores did not significantly differ. Despite these differences, in-hospital mortality did not significantly differ between immune phenotypes (39.0% vs 30.6%, *P* = 0.224), indicating that immune phenotyping captured biologically distinct host-response states without independently determining mortality.

**Table 2:** Clinical, laboratory, and immune characteristics of patients stratified by immune phenotype.

Variable	Immune-preserved (n=306)	Immune-suppressed (n=61)	Distribution	Test	Statistic	*P* value
Demographics						
Age (years)	66.14 ± 15.18	62.34 ± 15.71	Normal	Welch t	1.73	0.087
Sex	F101/M205	F11/M50	Categorical	Fisher	—	0.022
Severity scores						
APACHE II	24.62 ± 7.83	26.21 ± 7.46	Normal	Welch t	-1.51	0.135
SOFA	8.73 ± 3.28	10.07 ± 3.84	Normal	Welch t	-2.55	0.013
NUTRIC	6.41 ± 2.09	6.85 ± 1.71	Normal	Welch t	-1.78	0.078
Vital signs						
Temperature(°C)	36.8	36.7	Skewed	Mann U	1.11	0.264
Heart rate(beats/min)	100.53	101.79	Normal	Welch t	-0.32	0.751
MAP(mmHg)	64	64	Skewed	Mann U	0.81	0.42
Laboratory indicators						
PaO2/FiO2	262.19	265.1	Normal	Welch t	-0.17	0.863
Glucose(mmol/L)	9.38	9.92	Normal	Welch t	-0.95	0.344
Albumin(g/L)	30	30	Skewed	Mann U	0.07	0.944
BUN(mmol/L)	10.6	14.5	Skewed	Mann U	-3.15	0.002
Creatinine(umol/L)	117	127	Skewed	Mann U	-1.06	0.288
Bilirubin(umol/L)	19	23.7	Skewed	Mann U	-1.83	0.068
Platelets(×10^9^/L)	134.5	67	Skewed	Mann U	5.63	<0.001
Lactate(mmol/L)	1.6	1.9	Skewed	Mann U	-1.52	0.13
Hemoglobin(g/L)	92.89	92.44	Normal	Welch t	0.13	0.895
RDW-CV	14.8	15.6	Skewed	Mann U	-1.84	0.066
Procalcitonin(ng/mL)	4.2	11.8	Skewed	Mann U	-2.28	0.022
IL-6(pg/mL)	158.1	306	Skewed	Mann U	-2.28	0.022
CRP(mg/L)	151.02	190.33	Normal	Welch t	-2.14	0.036
WBC(×10^9^/L)	13.2	6.1	Skewed	Mann U	7.04	<0.001
Neutrophils(%)	89.1	92	Skewed	Mann U	-3.21	0.001
Monocytes(%)	4.2	2.3	Skewed	Mann U	3.76	<0.001
Lymphocytes(%)	6.2	3.4	Skewed	Mann U	4.48	<0.001
Immune indicators						
Total T cells(cells/uL)	431.6	95.6	Skewed	Mann U	11.5	<0.001
CD4+ T cells(cells/uL)	258	61.2	Skewed	Mann U	10.83	<0.001
CD8+ T cells(cells/uL)	149.6	30	Skewed	Mann U	10.84	<0.001
B cells(cells/uL)	110.5	16.8	Skewed	Mann U	8.59	<0.001
NK cells(cells/uL)	100.1	18	Skewed	Mann U	9.95	<0.001
HLA-DR(%)	53.75	40.98	Normal	Welch t	3.68	<0.001
Treatment						
Mechanical ventilation	2	4	Skewed	Mann U	-1.57	0.108
Thymosin	213/74	30/27	Categorical	Fisher	—	0.002
Ulinastatin	212/75	41/16	Categorical	Fisher	—	0.745
Outcome: mortality						
Mortality	91 (30.6%)	23 (39.0%)	Categorical	Fisher	—	0.224

Data are presented as mean ± standard deviation or median (interquartile range), unless otherwise indicated. Categorical variables are presented as number (percentage). APACHE II, Acute Physiology and Chronic Health Evaluation II; SOFA, Sequential Organ Failure Assessment; NUTRIC, Nutrition Risk in the Critically Ill; MAP, mean arterial pressure; BUN, blood urea nitrogen; RDW-CV, red blood cell distribution width coefficient of variation; PCT, procalcitonin; IL-6, interleukin-6; CRP, C-reactive protein; WBC, white blood cell; NK, natural killer; HLA-DR, human leukocyte antigen-DR. Statistical comparisons were performed using Welch’s t-test, Mann–Whitney U test, or Fisher’s exact test according to variable distribution and type.

### Prognostic information resides predominantly in conventional laboratory variables

**Figure 4:**
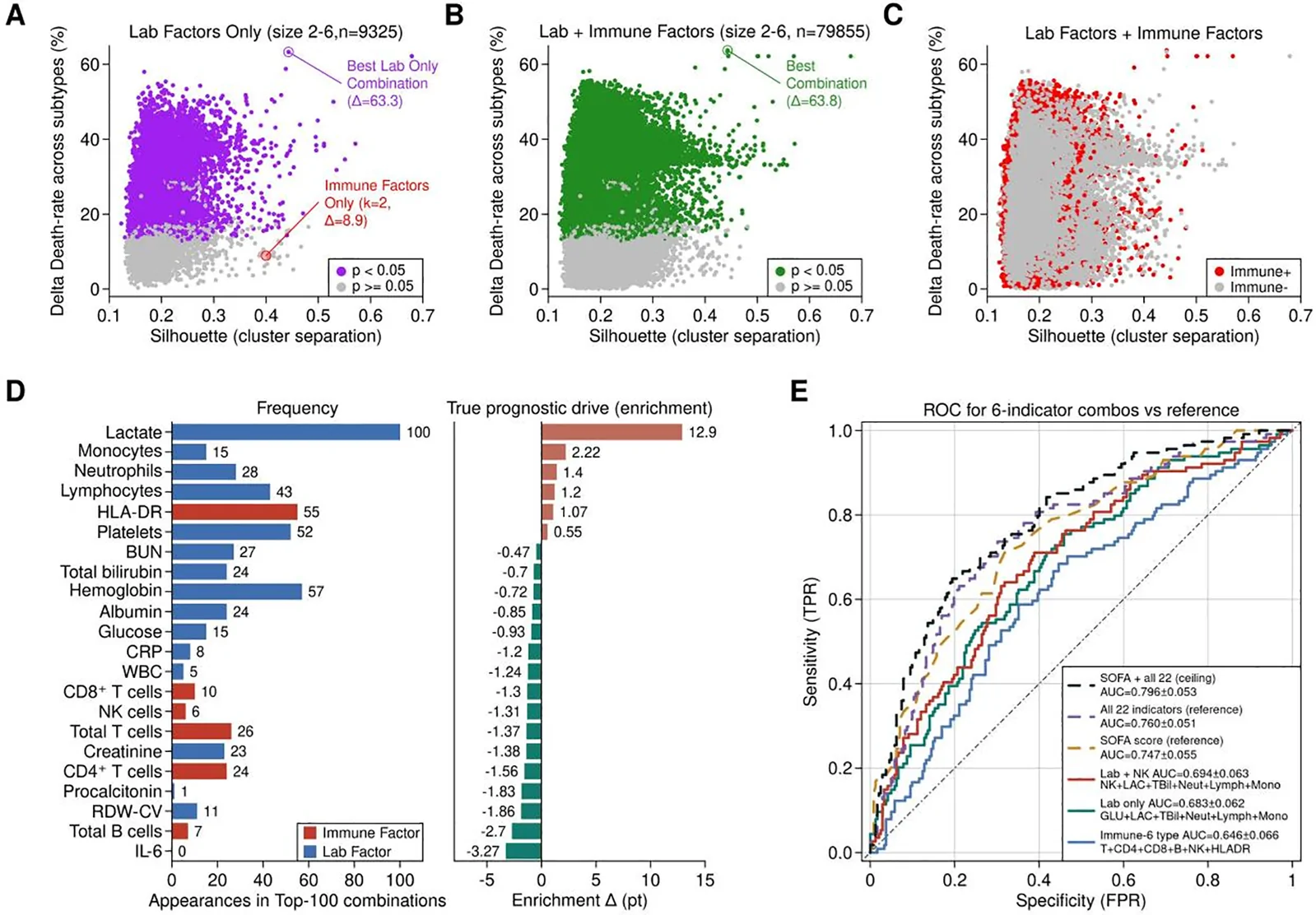
Prognostic benchmarking of immune phenotyping against routine clinical-laboratory variables. **(A)** Exhaustive combinatorial clustering using laboratory variables only. Each point represents one variable combination of size 2-6, plotted according to cluster separation and mortality-rate spread across clusters. Significant combinations are highlighted, and the best laboratory-only combination achieved a mortality-rate spread of 63.3 percentage points. The two-cluster immune-phenotype comparison is shown as a reference. **(B)** Exhaustive combinatorial clustering using the combined laboratory-plus-immune variable pool. The best-performing combined model achieved a mortality-rate spread of 63.8 percentage points. **(C)** Distribution of combined-pool clustering results stratified by whether the variable combination included immune markers. **(D)** Frequency and enrichment analysis of variables appearing in top-performing prognostic combinations. Lactate appeared most frequently and showed the strongest positive enrichment, whereas most lymphocyte-subset variables showed minimal or negative enrichment. **(E)** Receiver operating characteristic curves comparing repeated cross-validated logistic regression models based on immune markers, laboratory-marker combinations, SOFA, all combined indicators, and SOFA plus all indicators. The six-marker immune model showed the weakest discrimination, whereas SOFA and models incorporating broader clinical-laboratory information achieved higher discrimination.

To benchmark the prognostic resolution of immune phenotyping against routine laboratory variables, exhaustive combinatorial clustering was performed across all variable combinations (**Figures 4A–C**). Among laboratory-only combinations, the best-performing model achieved a larger mortality spread (Δ = 63.3 percentage points) than the two-cluster immune phenotype comparison (Δ = 8.9 points). Because the number of clusters differed between these analyses, this mortality-spread comparison should be interpreted cautiously. Therefore, predictive modelling using repeated cross-validation was additionally performed to provide a more standardized assessment of prognostic discrimination. Expanding the search to include both immune and laboratory variables yielded only a marginal improvement (Δ = 63.8 points), suggesting negligible additive prognostic value from immune markers. Frequency-versus-enrichment analysis further identified lactate as the dominant prognostic driver (Δ = +12.9 points), followed by monocyte percentage, neutrophil percentage, lymphocyte percentage, HLA-DR, and platelet count (**Figure 4D**). Among immune variables, only HLA-DR showed weak positive enrichment, whereas all lymphocyte subsets contributed minimally or negatively.

### Predictive modelling confirms limited incremental value of immune phenotyping

Cross-validated logistic regression models were constructed to evaluate predictive performance (**Figure 4E**). The six-marker immune model demonstrated the lowest discriminative ability (AUC = 0.646 ± 0.066), underperforming the best laboratory-based six-marker model (AUC = 0.683 ± 0.062). Substituting NK cells into the laboratory model did not significantly improve performance (AUC = 0.694 ± 0.063; ΔAUC = 0.008, *P* = 0.48), confirming minimal incremental value of immune variables. Notably, SOFA alone outperformed all six-marker models (AUC = 0.747 ± 0.055), while the full 22-variable model (AUC = 0.760 ± 0.051) and the ceiling model (SOFA + all variables; AUC = 0.796 ± 0.053) defined the upper performance boundary. Immune-only models demonstrated moderate discriminatory ability, whereas incorporation of immune markers into models containing established severity indicators resulted in limited additional improvement in discrimination. These findings suggest that immune profiling may provide biological stratification information but does not substantially enhance short-term mortality prediction beyond conventional clinical indicators in this cohort.

### Association between immune phenotype and illness severity

**Figure 5:**
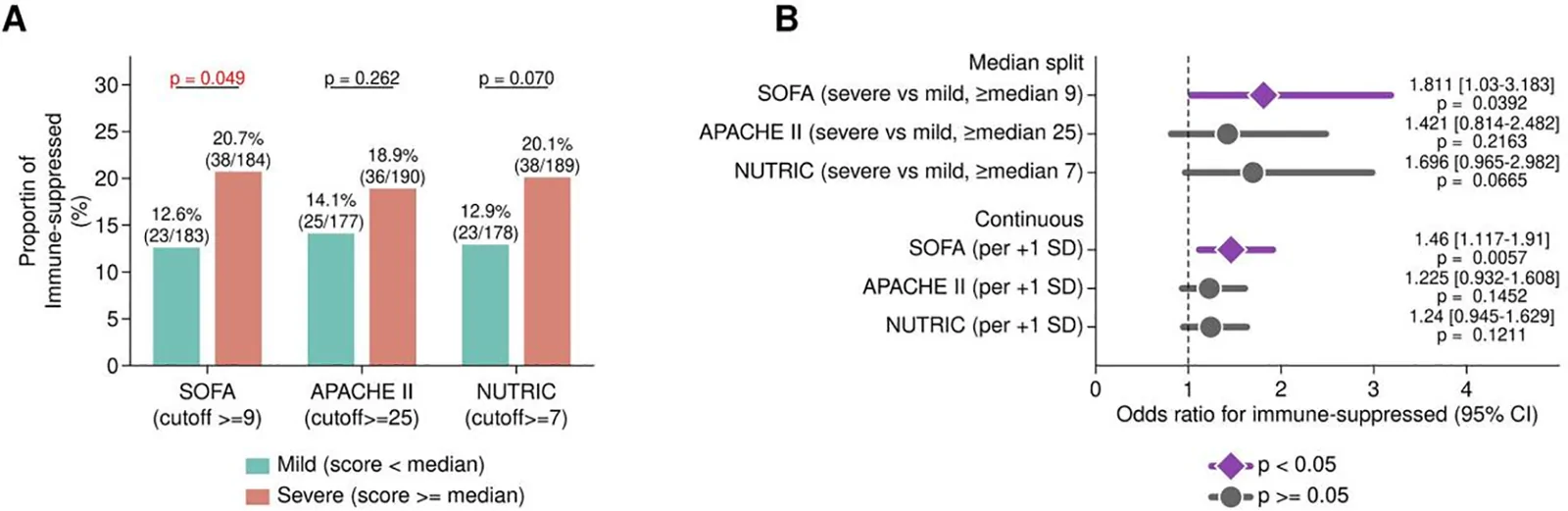
Association between immune phenotype and illness-severity scores. **(A)** Proportion of patients classified as immune-suppressed after stratification by median-split thresholds for SOFA, APACHE II, and NUTRIC scores. The immune-suppressed phenotype was more frequent among patients with higher SOFA scores, whereas the associations with APACHE II and NUTRIC were not statistically significant. **(B)** Univariable logistic regression analysis evaluating associations between illness-severity scores and the immune-suppressed phenotype. SOFA was significantly associated with the immune-suppressed phenotype both as a dichotomized median-split variable and as a continuous standardized predictor, whereas APACHE II and NUTRIC showed directionally consistent but non-significant associations. Odds ratios are shown with 95% confidence intervals.

The immune-suppressed phenotype was more frequently observed among patients with higher illness-severity scores, although associations were modest (**Figures 5A, B**). Using median-split analyses, only SOFA showed a statistically significant association (*P* = 0.049), whereas APACHE II and NUTRIC did not. In univariable logistic regression, all three scores showed directionally consistent positive associations with the immune-suppressed phenotype; however, only SOFA remained significant both as a dichotomized variable (OR 1.81, 95% CI 1.03–3.18, *P* = 0.039) and as a continuous predictor (OR 1.46 per +1 SD, 95% CI 1.12–1.91, *P* = 0.006). These findings indicate that immune phenotypes partially track disease severity but represent a distinct and only partially overlapping dimension of host-response biology.

## Discussion

In this study, we identified two internally stable immune phenotypes in sepsis in sepsis an immune-preserved phenotype and an immune-suppressed phenotype using a six-marker peripheral immune profiling framework. The immune-suppressed phenotype was characterized by globally reduced lymphocyte subsets and lower monocyte HLA-DR expression, consistent with the established biological concept of sepsis-induced immunoparalysis. However, despite clear immunological separation, immune phenotyping demonstrated limited incremental prognostic value for in-hospital mortality compared with conventional laboratory indicators and illness-severity scores. These findings suggest that peripheral immune phenotyping captures a distinct biological dimension of host-response heterogeneity but may be more valuable for immune stratification than for mortality prediction.

A major finding of this study was the identification of a distinct immunosuppressed endotype comprising 16.6% of the cohort. This observation aligns with previous transcriptomic and flow-cytometric studies demonstrating that a substantial subset of septic patients develops profound immune dysfunction characterized by lymphopenia, impaired antigen presentation, and monocyte deactivation (20, 21). Recent large-scale data from Monneret et al. showed that persistently low monocyte HLA-DR expression is strongly associated with ICU-acquired infections and adverse outcomes, supporting its role as a hallmark biomarker of sepsis immunoparalysis (13). Similarly, Samuelsen et al. demonstrated that HLA-DR and lymphocyte depletion remain among the most robust indicators of immune suppression in critically ill patients (18). Pyroptosis has been proposed in previous studies as one possible contributor to lymphocyte loss in sepsis; however, this mechanism was not directly assessed in the present study (22).

A highly inflammatory form of programmed cell death that has been shown to extensively consume lymphocytes during sepsis through inflammasome-mediated caspase-1/4/5/11 activation and subsequent gasdermin D-dependent membrane pore formation (23). This pathway represents a key mechanism of immune cell “self-sacrifice” in response to invasive pathogens, potentially contributing to the depletion of multiple lymphocyte subsets observed in our clustering analysis. Our findings extend these observations by showing that integrated clustering of multiple immune markers can resolve coherent host-response endotypes even using a simplified and clinically accessible panel.

An important biological feature of the immune-suppressed phenotype was the simultaneous elevation of inflammatory mediators, including IL-6, CRP, and PCT, despite substantial depletion of adaptive and innate immune cell populations. This finding supports the modern concept that hyperinflammation and immunosuppression coexist rather than occur as strictly sequential phases in sepsis (7). Meyer and Prescott are, emphasized that sepsis involves concurrent activation of pro-inflammatory and compensatory anti-inflammatory pathways, which collectively shape disease progression (24). Our data provide further clinical evidence for this dual-state model, indicating that immunosuppression should not be interpreted as immunological quiescence but rather as part of a broader dysregulated inflammatory network. Despite the clear biological distinction between the immune-preserved and immune-suppressed phenotypes, we observed no significant difference in in-hospital mortality. This finding contrasts with earlier evidence suggesting that impaired monocyte HLA-DR expression and lymphocyte depletion are associated with adverse outcomes and increased susceptibility to secondary infections (17). More recent studies, however, indicate that the prognostic utility of HLA-DR is highly dependent on its temporal trajectory rather than baseline assessment alone. In a large contemporary cohort (13),demonstrated that persistent suppression or delayed recovery of monocyte HLA-DR during ICU stay was more strongly associated with adverse outcomes than admission values. Likewise, longitudinal immune profiling studies have shown that immune recovery trajectories provide superior prognostic resolution compared with static single-time-point measurements (18). These observations may explain the limited prognostic discrimination observed in our cohort, where immune profiling was restricted to the early admission phase. Our findings support the concept that baseline immune phenotyping primarily captures the initial host-response state, whereas longitudinal immune monitoring may better reflect disease evolution and therapeutic responsiveness.

Our combinatorial clustering analyses demonstrated that conventional laboratory variables provided substantially greater prognostic resolution than immune markers alone. In particular, lactate emerged as the dominant prognostic clinical laboratory indicator determinant across high-performing combinations. This is consistent with extensive evidence showing lactate as one of the strongest predictors of mortality in sepsis, reflecting systemic hypoperfusion, metabolic stress, and mitochondrial dysfunction (6, 25, 26). The Surviving Sepsis Campaign continues to prioritize lactate as a core biomarker for risk stratification and therapeutic monitoring (27). The superior prognostic performance of lactate compared with immune variables suggests that early mortality risk in sepsis is more strongly determined by physiological collapse and organ dysfunction than by isolated immune status. Predictive modelling further reinforced this observation. The six-marker immune model demonstrated the weakest discrimination, whereas SOFA alone outperformed all six-marker models. This finding highlights the robust clinical utility of organ dysfunction-based severity scoring and indicates that immune phenotyping should complement rather than replace conventional prognostic tools. The mortality-spread comparison between immune and laboratory clustering approaches should be interpreted with caution because the clustering frameworks used different numbers of groups. Increasing cluster number may increase the separation between extreme outcome groups by design. Therefore, mortality spread alone should not be considered a definitive measure of prognostic superiority. The additional cross-validated prediction analyses were performed to provide a complementary evaluation of prognostic discrimination under a more standardized modelling framework. Instead, immune phenotyping may provide additional biological information not fully captured by SOFA or APACHE II. This interpretation is supported by recent precision immunotherapy studies, in which biomarker-guided host-directed interventions improved organ recovery and infection control despite inconsistent mortality benefits (28). These findings support an evolving role for immune profiling in therapeutic stratification rather than primary mortality prediction. Interestingly, the immune-suppressed phenotype showed only partial overlap with established illness-severity scores, with SOFA demonstrating the strongest association. This partial concordance suggests that immune dysfunction and organ dysfunction represent interconnected but biologically distinct dimensions of sepsis. While severity scores primarily quantify downstream organ failure, immune phenotyping may better reflect upstream immunological competence and vulnerability to secondary infections (9). This distinction may explain why immune markers add limited short-term prognostic value while retaining potential relevance for biological stratification and future investigation of individualized host-directed therapies. However, whether these immune phenotypes can predict response to immunomodulatory therapies remains unknown and requires prospective studies incorporating treatment-response assessment, longitudinal immune monitoring, and clinical outcomes. An important consideration is that several clustering variables represent related immune-cell populations and may partially reflect generalized lymphocyte depletion. The strong contribution of lymphocyte subsets to PC1 indicates that lymphocyte abundance represents a major component of the identified phenotype. However, the independent contribution of monocyte HLA-DR to PC2 and the coordinated reduction across both adaptive and innate immune compartments suggest that the immune-suppressed phenotype reflects a broader host-response pattern rather than lymphopenia alone. Future studies incorporating functional immune assays, cytokine profiles, and multi-omics approaches may further resolve the multidimensional structure of sepsis immune endotypes.

Several limitations of this study should be acknowledged. The single-center design enabled consistent clinical management, laboratory procedures, and immune assessment, providing a reliable framework for phenotype discovery; however, validation in larger multicenter cohorts is required to confirm generalizability across diverse populations.

This study focused on early baseline immune profiling to characterize initial host-response states. Future longitudinal studies incorporating serial immune monitoring, functional immune assays, cytokine profiling, and multi-omics approaches may further define the dynamic evolution and biological complexity of sepsis immune phenotypes. The six-marker immune panel was selected for translational feasibility and clinical accessibility, although additional biological measurements may improve immune characterization. Because immune profiling was performed as part of routine clinical assessment, the timing of sampling relative to early therapeutic interventions could not be completely standardized. Although samples were collected within 24 hours of admission whenever possible, some patients may have received initial treatments before immune assessment, and therefore the identified phenotypes should be interpreted as early clinical immune states rather than untreated baseline immune profiles. Although multiple clustering metrics were evaluated, some variability existed regarding the optimal number of clusters. The final two-cluster solution was selected based on statistical characteristics, biological interpretability, and clinical relevance. These immune phenotypes represent internally stable patterns within the current cohort, and independent multicenter validation is required to establish their broader reproducibility. Finally, although repeated cross-validation was applied during predictive modelling, the best-performing laboratory marker combination was selected before model evaluation; therefore, predictive estimates should be interpreted as exploratory. Formal nested model comparisons evaluating the incremental prognostic contribution of immune markers beyond SOFA and routine laboratory indicators were not performed and should be addressed in future studies.

## Conclusion

Peripheral immune phenotyping using a simplified six-marker panel identifies biologically distinct immune-preserved and immune-suppressed states in sepsis, reflecting important dimensions of host immune dysregulation. Although these immune phenotypes provide limited additional prognostic discrimination for in-hospital mortality beyond established severity scores and conventional laboratory indicators, they offer valuable insights into immunoparalysis and host-response heterogeneity. These findings support the potential role of immune phenotyping as a complementary approach for biological stratification and longitudinal immune monitoring, while prospective multicenter studies are required to determine its clinical utility in guiding personalized immunomodulatory strategies.

## Data Availability

The raw data supporting the conclusions of this article will be made available by the authors, without undue reservation.
