# Supplementary material for: Peripheral immune phenotyping identifies an immunosuppressed sepsis endotype with potential relevance for immune stratification
Source: Front Immunol. 2026 Sep 4;17:1939205. doi: 10.3389/fimmu.2026.1939205 (PMC13585658; doi:10.3389/fimmu.2026.1939205)
Supplement: Supplementary file 1 [file DataSheet1.pdf]

frontiers

The International Society for the Advancement of Cytometry (ISAC) has highlighted the importance of including comprehensive methodological information to ensure data reproducibility and reliability. In line with this, Frontiers in Immunology now requires authors to submit a checklist for manuscripts that involve flow or mass cytometry. This checklist helps standardize the reporting process, improving the quality and transparency of published data. By doing so, we support scientific progress, making it easier for other researchers to replicate and validate experiments.

This form should be submitted with any manuscripts using flow or mass cytometry.

Sample/specimen/material description

☑ Total blood

☐ PBMCs

☐ Organ digests

Other____

Did the samples suffer any treatment before or after incubation with the antibodies?

☐ Drug ____________________________________

☐ Cell permeabilization _______________________

☐ Dye _____________________________________

☐ Propidium iodine

☐ Not applicable

Other RBC lysis

Instrument and antibodies

Name of the Cytometer BD FACSCanto II

Antibodies and targets	Fluorochrome/ Metal	Catalog number/Company
e.g. anti-CD4	FITC	Cat. XXX/ XXX ltd.
anti-CD3	FITC	Cat.662967/BD Biosciences

anti-CD4	PE-Cy7	Cat.662967/BD Biosciences
anti-CD8	APC-Cy7	Cat.662967/BD Biosciences
anti-CD19	APC	Cat.662967/BD Biosciences
anti-CD16+anti-CD56	PE	Cat.662967/BD Biosciences
anti-HLA-DR+anti-CD14	APC(HLA-DR) FITC(CD14)	Biosciences; Cat.665753(CD14)/BD Biosciences

Data analyses

1. Name of the software MRFlow and BD FACSCanto II
2. Reference gating strategy in the manuscript or supplementary material

Gating strategy in (eg Figure X) Gating strategy shown in Supplementary Figure S1 and S2
