# Supplementary material for: Peripheral immune phenotyping identifies an immunosuppressed sepsis endotype with potential relevance for immune stratification
Source: Front Immunol. 2026 Sep 4;17:1939205. doi: 10.3389/fimmu.2026.1939205 (PMC13585658; doi:10.3389/fimmu.2026.1939205)
Supplement: Supplementary file 2 [file DataSheet2.docx]

***Supplementary Material***

**Supplementary Table S1.** Detailed characteristics of antibodies, clones, fluorochromes, and target immune-cell populations used for multiparameter flow cytometry analysis.

| **Marker/Population** | **Antibody** | **Clone** | **Fluorochrome** | **Purpose/Identification** |
| --- | --- | --- | --- | --- |
| Total T cells | CD3 | SK7 | FITC | Identification of CD3⁺ T cells |
| CD4 T cells | CD3/CD4 | SK7/SK3 | PE-Cy7 | Identification of CD4⁺ T cells |
| CD8 T cells | CD3/CD8 | SK7/SK1 | APC-Cy7 | Identification of CD8⁺ T cells |
| B cells | CD19 | SJ25C1 | APC | Identification of B cells |
| NK cells | CD3/CD16/CD56 | SK7/B73.1/NCAM16.2 | PE | Identification of NK cells |
| Monocyte HLA-DR | HLA-DR | L243 | APC | Assessment of monocyte activation status |

**Supplementary Figure S1. Representative Flow cytometry dot plots for absolute counting of peripheral blood lymphocyte subsets.**

Notes: Representative hierarchical gating strategy for peripheral blood lymphocyte subsets. First, lymphocytes were gated according to CD45 expression and side scatter (SSC). Within the lymphocyte population, T cells (CD3⁺) and non-T cells (CD3⁻) were separated. CD3⁺ T lymphocytes were further subdivided into CD4⁺ and CD8⁺ T cell subsets. In the CD3⁻ non-T cell compartment, CD19⁺ B cells and CD16/56⁺ NK cells were identified.

**Figure S2. Representative Flow cytometry dot plots showing HLA-DR expression on peripheral blood monocytes.**

Notes: Cell populations were initially distinguished by forward scatter (FSC-A) and side scatter (SSC-A). CD14⁺ monocytes were subsequently gated using CD14-FITC and SSC-A. Within the monocyte gate, CD14 and HLA-DR expression were analyzed to identify HLA-DR-positive and HLA-DR-negative monocytes for evaluation of monocytic HLA-DR (mHLA-DR).
